# Challenges and Strategies to Maintain Fidelity to the MIRROR-TCM Intervention During the COVID-19 Pandemic

**DOI:** 10.1093/geroni/igab046.903

**Published:** 2021-12-17

**Authors:** Mary Naylor, Karen Hirschman, Brianna Morgan, Molly McHugh, Elizabeth Shaid, Kathleen McCauley, Christina Whitehouse, Mark Pauly

**Affiliations:** 1 University of Pennsylvania, Philadelphia, Pennsylvania, United States; 2 Villanova University, Villanova Univesity, Pennsylvania, United States

## Abstract

Randomized clinical trials (RCTs) have demonstrated that the multicomponent Transitional Care Model (TCM), an advanced practice registered nurse-led, team-based, care management strategy improves outcomes for older adults transitioning from hospital to home. However, healthcare systems’ adoption of the model has been limited. A multi-system, replication RCT (MIRROR-TCM) enrolling older adults hospitalized with heart failure, chronic obstructive pulmonary disease or pneumonia began in February 2020 just as the outbreak of COVID-19 in the U.S. dramatically changed the healthcare and research landscape. The goal of this qualitative descriptive study is to explore the impact of COVID-19 on fidelity to the TCM intervention during this clinical trial. Using directed content analysis, recorded monthly meetings with health system leaders and staff were coded to identify challenges and strategies to maintaining fidelity to the intervention in the context of the pandemic. Analyses showed that COVID-19 impacted all 10 TCM components. The components with the most challenges were delivering services from hospital-to-home due to quarantining, restrictive facility policies, lack of personal protective equipment and limited telehealth availability; coordinating care due reduced availability of services, and screening at risk individuals because of fewer eligible patients. Strategies for addressing challenges included: exploring alternatives (e.g., increasing reliance on telehealth, expanding study eligibility), building and engaging networks (e.g., direct outreach to skilled nursing facility staff) and anticipating needs (e.g., preparing for shorter hospital stays). Findings highlight the importance of monitoring the contextual challenges to implementing an evidence-based intervention and actively engaging partners in identifying strategies to achieve fidelity.

